# Gene expression fingerprint of uterine serous papillary carcinoma: identification of novel molecular markers for uterine serous cancer diagnosis and therapy

**DOI:** 10.1038/sj.bjc.6602480

**Published:** 2005-03-22

**Authors:** A D Santin, F Zhan, S Cane', S Bellone, M Palmieri, M Thomas, A Burnett, J J Roman, M J Cannon, J Shaughnessy, S Pecorelli

**Affiliations:** 1 Department of Obstetrics & Gynecology, Division of Gynecologic Oncology, University of Arkansas for Medical Sciences, Little Rock, AR, USA; 2 Myeloma Institute for Research and Therapy, University of Arkansas for Medical Sciences, Little Rock, AR, USA; 3 Department of Pathology, University of Arkansas for Medical Sciences, Little Rock, AR, USA; 4 Department of Microbiology & Immunology, University of Arkansas, Little Rock, AR, USA; 5 Division of Gynecologic Oncology, University of Brescia, Brescia, Italy

**Keywords:** serous papillary uterine cancer, gene expression profiling

## Abstract

Uterine serous papillary cancer (USPC) represents a rare but highly aggressive variant of endometrial cancer, the most common gynecologic tumour in women. We used oligonucleotide microarrays that interrogate the expression of some 10 000 known genes to profile 10 highly purified primary USPC cultures and five normal endometrial cells (NEC). We report that unsupervised analysis of mRNA fingerprints readily distinguished USPC from normal endometrial epithelial cells and identified 139 and 390 genes that exhibited >5-fold upregulation and downregulation, respectively, in primary USPC when compared to NEC. Many of the genes upregulated in USPC were found to represent adhesion molecules, secreted proteins and oncogenes, such as *L1 cell adhesion molecule*, *claudin-3* and *claudin-4*, *kallikrein 6* (*protease M*) and *kallikrein 10* (*NES1*), *interleukin-6* and *c-erbB2*. Downregulated genes in USPC included *SEMACAP3*, *ras homolog gene family*, *member I* (*ARHI*), and *differentially downregulated in ovarian carcinoma gene 1*. Quantitative RT–PCR was used to validate differences in gene expression between USPC and NEC for several of these genes. Owing to its potential as a novel therapeutic marker, expression of the high-affinity epithelial receptor for *Clostridium perfringens* enterotoxin (CPE) *claudin-4* was further validated through immunohistochemical analysis of formalin-fixed paraffin-embedded specimens from which the primary USPC cultures were obtained, as well as an independent set of archival USPC specimens. Finally, the sensitivity of primary USPC to the administration of scalar doses of CPE *in vitro* was also demonstrated. Our results highlight the novel molecular features of USPC and provide a foundation for the development of new type-specific therapies against this highly aggressive variant of endometrial cancer.

Uterine cancer is the most prevalent gynecologic tumour in women, with an estimated 40,100 cases and 6800 deaths in the United States in 2003 (Jemal *et al*, 2003). On the basis of clinical and histopathologic variables, two subtypes of endometrial carcinoma, namely Type I and Type II tumours, have been described (Bohkman, 1983). Type I endometrial cancers, which account for the majority (i.e., about 80%) of cases, are usually well differentiated and endometrioid in histology. These neoplasms are frequently diagnosed in younger women, are associated with a history of hyperestrogenism as the main risk factor, and typically have a favourable prognosis with appropriate therapy. Type II endometrial cancers are poorly differentiated tumours, often with serous papillary or clear cell histology, and are not associated with hyperestrogenic factors. Although Type II tumours account for only a minority of endometrial carcinoma, about 50% of all relapses occur in this group of patients.

High-throughput technologies for assaying gene expression, such as high-density oligonucleotide and cDNA microarrays, have recently been used in an attempt to define the genetic fingerprints of a variety of human tumours including endometrial cancers (Smid-Koopman *et al*, 2000; Matsushima-Nishiu *et al*, 2001; Moreno-Bueno *et al*, 2003; Risinger *et al*, 2003). These techniques may allow precise and accurate grouping of human tumours and have the potential to identify patients who are unlikely to be cured by conventional therapy (Sorlie *et al*, 2001; Rosenwald *et al*, 2002; Moreno-Bueno *et al*, 2003; Risinger *et al*, 2003). Consistent with this view, clinically relevant genes highly differentially expressed between Type I and Type II endometrial tumours have recently been identified (Moreno-Bueno *et al*, 2003; Risinger *et al*, 2003). Most of the differentially expressed genes in Type I tumours included genes known to be hormonally regulated during the menstrual cycle and known to be important in endometrial homeostasis (i.e., *MGB2*, *LTF*, *END1*, and *MMP11*) (Moreno-Bueno *et al*, 2003; Risinger *et al*, 2003). In contrast, Type II tumours have been shown to overexpress genes involved in the regulation of the mitotic spindle checkpoint and associated with aneuploidy and an aggressive phenotype, such as *STK15*, *BUB1*, and *CCNB2* (Moreno-Bueno *et al*, 2003; Risinger *et al*, 2003).

Uterine serous papillary tumors (USPC) represent the most aggressive variant of Type II endometrial cancer and may constitute up to 10% of endometrial tumours. The microscopic criteria for diagnosis of USPC were first outlined by Hendrickson *et al* (1982). Pleomorphism, grade III nuclear atypia with prominent nucleoli and vesicular chromatin pattern, as well as a high mitotic activity are commonly detected in this tumour. Clinically, USPC has a propensity for early intra-abdominal and lymphatic spread even at presentation and is characterised by a highly aggressive biologic behaviour (Hendrickson *et al*, 1982; Nicklin and Copeland, 1996). Unlike the histologically indistinguishable high-grade serous ovarian carcinomas, USPC is a chemoresistant disease from onset, with responses to combined cisplatinum-based chemotherapy in the order of 20% and of short duration (Nicklin and Copeland, 1996). The overall 5-year survival is about 30% for all stages and the recurrence rate after surgery is extremely high (50–80%). A deeper understanding of the molecular basis of the aggressive biologic behaviour of USPC as well as the development of novel, more specific and more effective treatment modalities against this variant of endometrial cancer remain a high priority.

In this study, with the goal of identifying genes with a differential pattern of expression between USPC and normal endometrial cells (NEC) and to use this knowledge for the development of novel diagnostic and therapeutic markers against this disease, we used oligonucleotide microarrays which interrogate the expression of some 10 000 known genes to analyse gene expression profiling of 10 highly purified primary USPC cultures and five primary NEC. We report a number of genes which may readily distinguish USPC from normal endometrial epithelial cells. More importantly, these results highlight the novel molecular features of USPC and provide a foundation for the development of new type-specific diagnostic and therapeutic strategies for this disease.

## MATERIALS AND METHODS

### Establishment of USPC and NEC primary cell lines

A total of 15 primary cell lines (i.e., 10 USPC and five NEC) were established after sterile processing of samples from surgical biopsies collected between 1997 and 2004 at the University of Arkansas for Medical Sciences, as previously described for USPC specimens (Santin *et al*, 2002) and NEC cultures (Bongso *et al*, 1988). All fresh samples were obtained with appropriate consent according to IRB guidelines. Tumours were staged according to the FIGO operative staging system. A total abdominal hysterectomy with bilateral salpingo oophorectomy and bilateral pelvic lymphadenectomy was performed in all uterine carcinoma patients, while normal endometrial tissue was obtained from consenting donors undergoing surgery for benign pathology. No patient received chemotherapy or radiation before surgery. The patient characteristics are described in Table 1. Tumour cells were collected for RNA extraction at a confluence of 50–80% after a minimum of two to a maximum of 10 passages *in vitro*. The epithelial nature and the purity of USPC and NEC cultures were verified by immunohistochemical staining and flow-cytometric analysis with antibodies against cytokeratin and vimentin as described previously (Bongso *et al*, 1988; Santin *et al*, 2002). Only primary cultures which had at least 90% viability and contained >99% epithelial cells were used for total RNA extraction.

### RNA purification and microarray hybridization and analysis

Detailed protocols for RNA purification, cDNA synthesis, cRNA preparation, and hybridization to the Affymetrix Human U95Av2 GeneChip microarray were performed according to the manufacturer's protocols, as reported previously (Zhan *et al*, 2002).

### Data processing

All data used in our analyses were derived from Affymetrix 5.0 software. GeneChip 5.0 output files are given as a signal that represents the difference between the intensities of the sequence-specific perfect match probe set and the mismatch probe set, or as a detection of present, marginal, or absent signals as determined by the GeneChip 5.0 algorithm. Gene arrays were scaled to an average signal of 1500 and then analysed independently. Signal calls were transformed by the log base 2 and each sample was normalized to give a mean of 0 and variance of 1.

### Gene expression data analysis

Statistical analyses of the data were performed with the software packages SPSS10.0 (SPSS, Chicago, IL) and the significance analysis of microarrays (SAM) method (Tusher *et al*, 2001). Genes were selected for analysis based on detection and fold change. In each comparison, genes having ‘present’ detection calls in more than half of the samples in the overexpressed gene group were retained for statistical analysis if they showed >5-fold change between groups. Retained genes were subjected to SAM to establish a false discovery rate (FDR), then further filtered via the Wilcoxon rank sum (WRS) test at alpha=0.05. The FDR obtained from the initial SAM analysis was assumed to characterise genes found significant via WRS.

### Gene cluster/treeview

The hierarchical clustering of average-linkage method with the centred correlation metric was used (Eisen *et al*, 1998). For the unsupervised hierarchical clustering, a total of 7328 probe sets were scanned across 10 USPCs and five NECs. The 7328 probe sets were derived from 12 588 by filtering out all control genes, all genes with absent detections, and genes not fulfilling the test of standard deviation greater than 0.5 (0.5 being the log base 2 of the signal). Only genes significantly expressed by both WRS and SAM analyses and whose average change in expression level was at least five-fold are shown in the Results section.

### Quantitative real-time PCR (q-RT–PCR)

Quantitative real-time PCR was performed with an ABI Prism 7000 Sequence Analyzer using the manufacturer's recommended protocol (Applied Biosystems, Foster City, CA, USA) to validate differential expression of selected genes in samples from all 15 primary cell lines (10 USPC and five NEC). Each reaction was run in triplicate. The comparative threshold cycle (*C*_T_) method was used for the calculation of amplification fold as specified by the manufacturer. Briefly, 5 *μ*g of total RNA from each sample was reverse transcribed using SuperScript II Rnase H Reverse Transcriptase (Invitrogen, Carlsbad, CA, USA). In all, 10 *μ*l of reverse-transcribed RNA samples (from 500 *μ*l of total volume) was amplified by using the TaqMan Universal PCR Master Mix (Applied Biosystems) to produce PCR products specific for *cyclin-dependent kinase inhibitor 2A* (*CDKN2A/p16* and *CDKN2A/p14ARF*), *L1 cell adhesion molecule* (*L1CAM*), *claudin-3*, *claudin-4*, *GRB7*, and *c-erbB2*. Primers specific for 18s ribosomal RNA and empirically determined ratios of 18s competimers (Applied Biosystems) were used to control for the amounts of cDNA generated from each sample. Sequences for primers and probes are available on request. Differences among USPC and NEC in the q-RT–PCR expression data were tested using the Kruskal–Wallis nonparametric test. Pearson product–moment correlations were used to estimate the degree of association between the microarray and q-RT–PCR data.

### Claudin-4 immunostaining of formalin-fixed tumour tissues

Claudin-4 protein expression was evaluated by immunohistochemical staining on formalin-fixed tumour tissue from which primary cultures were obtained. In addition, to further confirm transcriptional profiling results of USPC, claudin-4 marker was also evaluated by immunohistochemistry in a second independent set of eight USPC clinical tissue samples obtained from patients harbouring advanced stage disease (i.e., stage III and IV) treated at the UAMS during the same period. Study blocks were selected after histopathologic review by a surgical pathologist. The most representative haematoxylin and eosin-stained block sections were used for each specimen. Briefly, immunohistochemical stains were performed on 4-*μ*m-thick sections of formalin-fixed, paraffin-embedded tissue. After pretreatment with 10 mM citrate buffer at pH 6.0 using a steamer, they were incubated with mouse anti-claudin-4 antibodies (cat. #: 18-7341; Zymed Laboratories Inc., San Francisco, CA, USA) at 1 : 2000 dilution. Antigen-bound primary antibodies were detected using standard avidin–biotin immunoperoxidase complex (Dako Corp., Carpinteria, CA, USA). Cases with less than 10% staining in tumour cells were considered negative for claudin expression, while positive cases were classified as follows regarding the intensity of claudin-4 protein expression: (a) +, focal membrane staining; (b) ++, diffuse membrane staining; and (c) +++, diffuse membrane and cytoplasmic staining.

### *Clostridium perfringens* enterotoxin (CPE) treatment of primary USPC cell lines and trypan blue exclusion test

Tumour samples obtained from three patients harbouring advanced USPC (i.e., USPC 1, USPC 2, and USPC 3) and two NEC cultures derived from similar-aged women were seeded at a concentration of 1 × 10^5^ cells well^−1^ into six-well culture plates (Costar, Cambridge, MA, USA) with the appropriate medium. Tumour samples and control cell lines were grown to 80% confluence. After washing and renewal of the medium, recombinant CPE cloned and purified as previously described (Michl *et al*, 2001) was added to final concentrations ranging from 0.03 to 3.3 *μ*g ml^−1^. After incubation for 60 min to 24 h at 37°C, 5% CO_2_, floating cells were removed and stored, and attached cells were trypsinised and pooled with the floating cells. After staining with trypan blue, viability was determined by counting the number of trypan blue-positive cells and the total cell number.

## RESULTS

### Gene expression profiles distinguish USPC from NEC and identify differentially expressed genes

**Figure 1:**
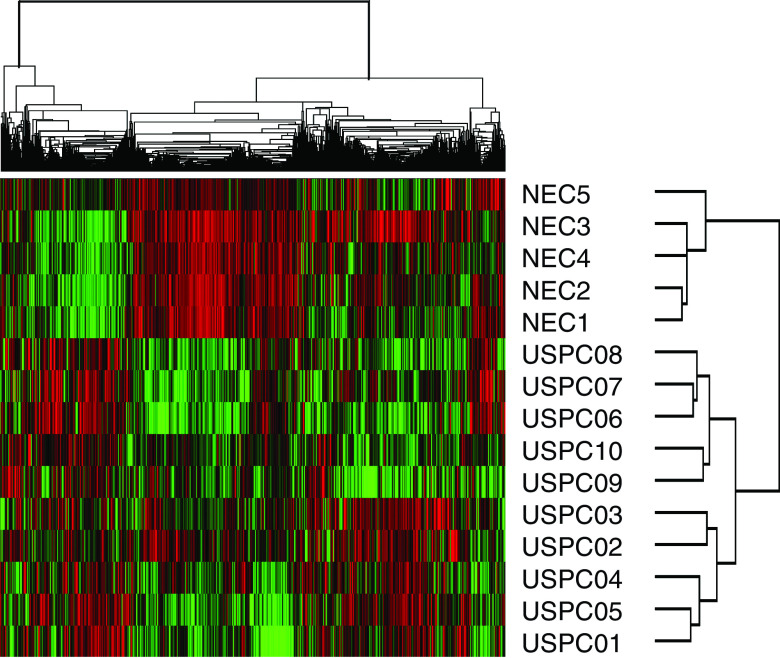
Unsupervised hierarchical clustering of 15 primary uterine cell lines (i.e., 10 USPC and five NEC). The cluster is colour coded using *red* for upregulation, *green* for downregulation, and *black* for median expression. Agglomerative clustering of genes is illustrated with dendrograms.

To minimise the risk of contamination of USPC RNA with that of normal cells or tumour cells with different histology (i.e., endometrioid or clear cells), as well as to reduce the complexity of gene expression data analysis, in this study we extracted RNA from short-term primary tumour cell cultures collected only from USPC with single-type differentiation (i.e., pure USPC). Short-term USPC and NEC cell cultures, minimising the risk of a selection bias inherent in any long-term *in vitro* growth, may provide an opportunity to study differential gene expression between highly enriched populations of normal and tumour-derived epithelial cells. Accordingly, comprehensive gene expression profiles of 10 primary USPC and five primary NEC cell lines were generated using high-density oligonucleotide arrays with 12 588 probe sets, which in total interrogated some 10 000 genes. Using unsupervised hierarchical cluster analysis with 7238 probe sets, we identified differences in gene expression between USPC and NEC, which readily distinguished the two groups of primary cultures. As shown in Figure 1, all 10 USPC were found to group together in the rightmost columns of the dendrogram. Similarly, in the leftmost columns, all five NEC were found to cluster tightly together. After filtering out most ‘absent’ genes, the SAM and the nonparametric WRS test (*P*<0.05) were performed to identify genes differentially expressed between USPC and NEC. A total of 2829 probe sets were found differentially expressed between USPC and NEC with *P*<0.05 by WRS and with a median FDR of 0.35% and a 90th percentile FDR of 0.59% by SAM. Of the 2829 aforementioned probe sets, there were 529 probe sets showing >5-fold change. As shown in Table 2, a group of 139 probe sets were found highly expressed in USPC and underexpressed in NEC. Included in this group of genes are *CDKN2A/p16/p14ARF* (101-fold), *L1CAM* (25-fold), *claudin-3* (eight-fold), and *claudin-4* (12-fold), *kallikrein 6* (*protease M*) (19-fold), *and kallikrein 10 (NES1)* (23-fold), *interleukin-6* (19-fold), *interleukin-18* (10-fold), and *plasminogen activator receptor* (*PLAUR*) (seven-fold) (Table 2). Importantly, *c-erbB2*, a gene recently found by our group to be highly differentially expressed in USPC when compared to ovarian serous papillary tumours (Santin *et al*, 2004), was 14-fold more highly expressed in USPC than in NEC (Table 2). The second profile was represented by 390 genes that were highly expressed in NEC and underexpressed in USPC. Table 3 depicts the genes showing >10-fold change. Included in this group of genes are *transforming growth factor beta receptor III*, *platelet-derived growth factor receptor alpha*, *SEMACAP3*, *ras homolog gene family*, *member I* (*ARHI*), and *differentially downregulated in ovarian carcinoma 1* (*DOC1*).

### Validation of the microarray data

**Figure 2:**
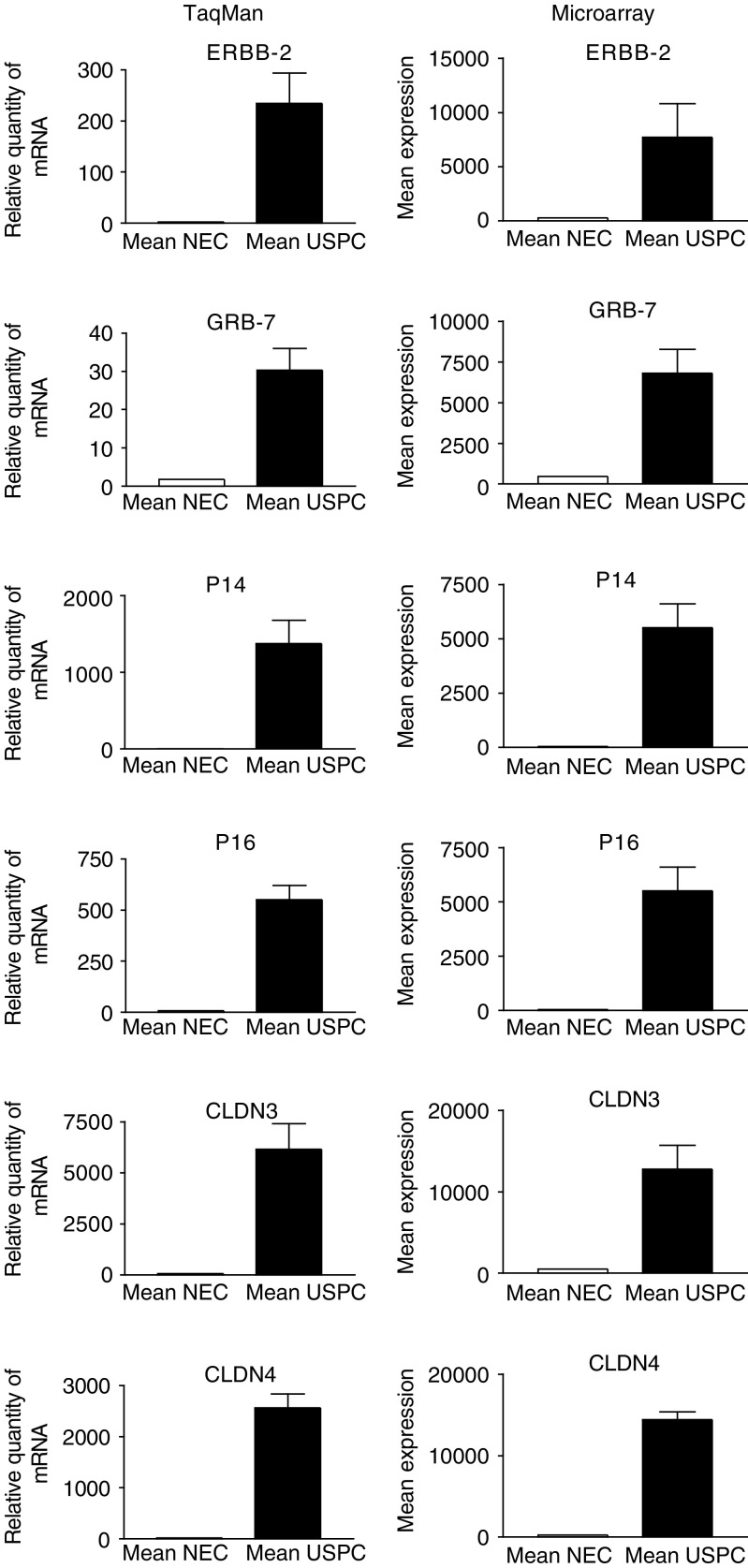
Quantitative RT–PCR and microarray expression analysis of *CDKN2A/p16*, *CDKN2A/p14ARF*, *claudin-3*, *claudin-4*, *GRB-7* and *c-erbB2* genes differentially expressed between USPC and NEC. Quantitative RT-PCR data were highly correlated to the microarray data (*P*<0.001).

We used q-RT–PCR assays to validate the microarray data. Seven highly differentially expressed genes between USPC and NEC (i.e., *CDKN2A/p16, CDKN2A/p14ARF* (101-fold), *L1CAM* (25-fold), *claudin-3* (eight-fold), *claudin-4* (12-fold), *GRB-7* (19-fold), and *c-erbB2* (14-fold)) were selected for q-RT–PCR analysis. A comparison of the microarray and q-RT–PCR data for six of these genes is shown in Figure 2. Expression differences between USPC and NEC for *CDKN2A/p16* (*P*=0.002), *CDKN2A/p14ARF* (*P*=0.002), *claudin-3* (*P*=0.01), *claudin-4* (*P*=0.002), *GRB-7* (*P*=0.002) and *c-erbB2* (*P*=0.01) were readily apparent (Table 2 and Figure 2). Moreover, for all seven genes tested, the q-RT–PCR data were highly correlated to the microarray data (*P*<0.001) (*r*=0.81, 0.80, 0.75, 0,69, 0.82, 0.71 and 0.65, respectively), with all the samples (i.e., 10 USPC and five NEC) included in both the q-RT–PCR and microarray experiments. Thus, q-RT–PCR data suggest that most array probe sets are likely to accurately measure the levels of the intended transcript within a complex mixture of transcripts.

### Claudin-4 expression by immunohistology on USPC and NEC tissue blocks

**Figure 3:**
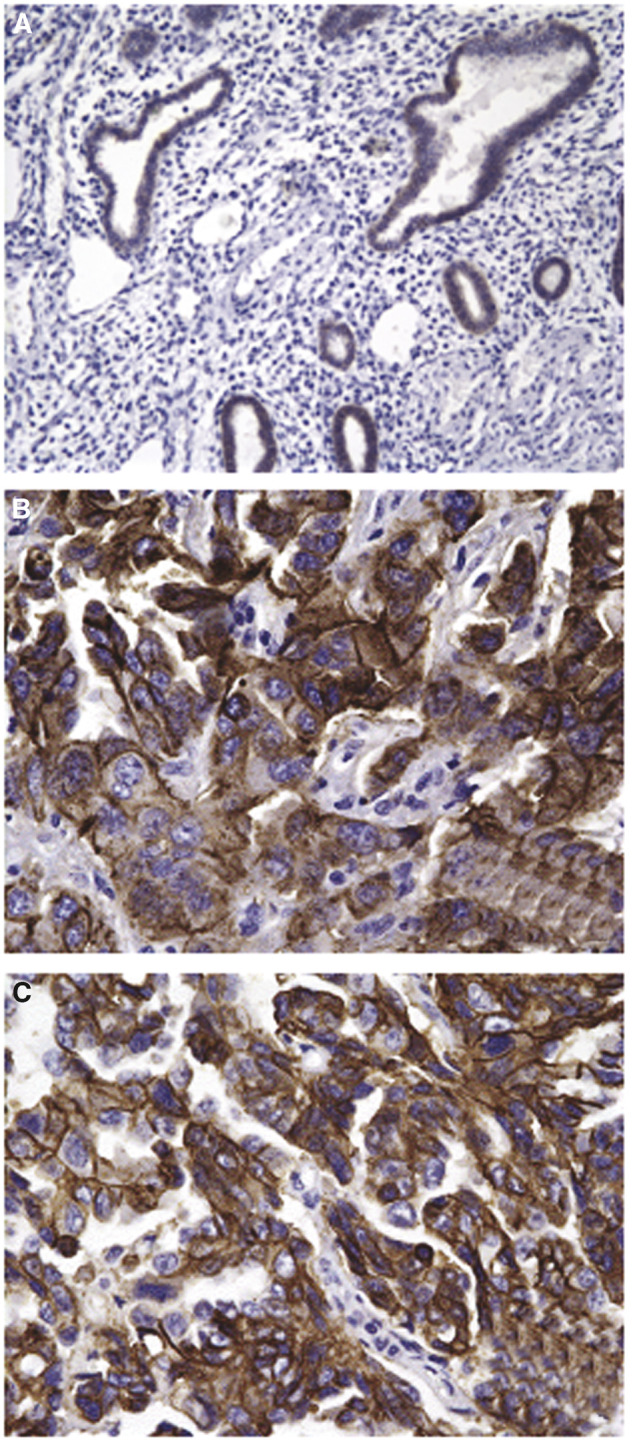
Representative immunohistochemical staining for claudin-4 on two paraffin-embedded USPC specimens (**B**–**C**) and one NEC specimen (**A**). Normal endometrial cell 1 (upper panel) showed light membrane staining for claudin-4, while USPC 1 and USPC 3 showed heavy cytoplasmic and membranous staining for claudin-4 (middle and lower panel). Note the large difference in the size of USPC cells when compared to the control glandular epithelium of a menopausal women. Original magnification × 400.

To determine whether the high or low expression of the *claudin-4* gene detected by microarray and q-RT–PCR assays in primary USPC and NEC cell lines, respectively, is the result of a selection of a subpopulation of cancer cells present in the original tumour, or whether *in vitro* expansion conditions may have modified gene expression, we performed immunohistochemical analysis of claudin-4 protein expression on formalin-fixed tumour tissue from the uncultured primary surgical specimens from which the USPC cell lines were derived. As shown in Table 4 and representatively in Figure 3, both cytoplasmic and membranous staining for claudin-4 protein expression was noted in the majority of USPC specimens (i.e., 90% score 3+ and 2+). In contrast, only low levels of membranous staining for claudin-4 protein was found in the NEC tissue samples tested by immunohistochemistry (Table 4, Figure 3, *P*=0.02 USPC *vs* NEC by Student *t*-test). To confirm and validate the immunohistochemical results in an independent series of USPC, formalin-fixed tumour tissue blocks from eight further surgical specimens (i.e., USPC 11–18, Table 4) similarly obtained from patients harbouring advanced stage disease were tested for claudin-4 expression. Again, heavy cytoplasmic and/or membranous staining for the claudin-4 receptor was found in the striking majority of the further USPC sample tested.

### Effects of CPE on primary USPC and normal control cells

**Figure 4:**
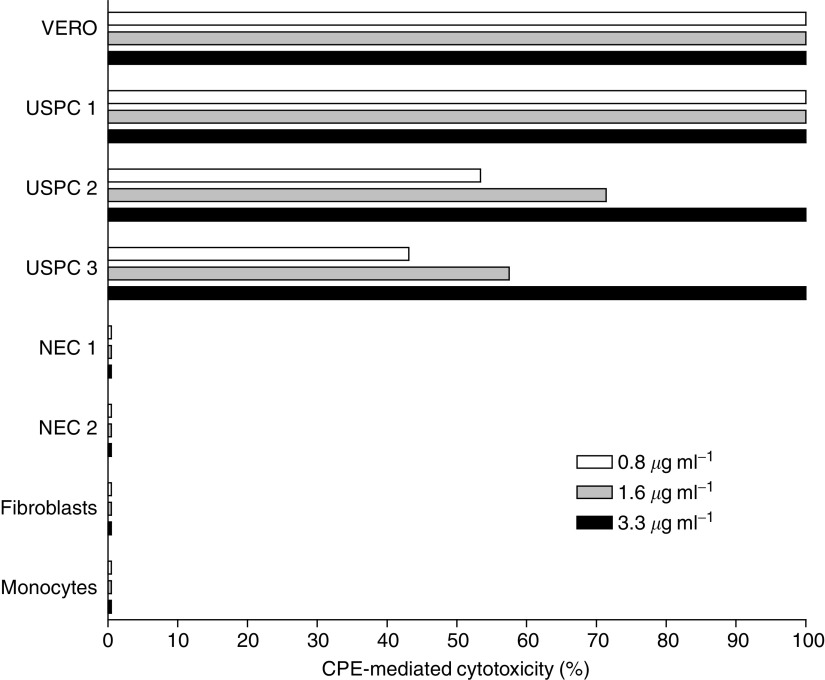
Representative dose-dependent CPE-mediated cytotoxicity of primary USPC compared to positive control Vero cells or negative controls (i.e., NEC, fibroblasts, and monocytes) after 24 h exposure to scalar doses of CPE. VERO, positive control cells. Uterine serous papillary carcinoma-1 to USPC-3 primary uterine serous tumors. NEC, normal endometrial cells. Fibroblasts, normal human fibroblasts. Monocytes, normal mononuclear cells.

The sensitivity of primary uterine serous tumour cultures to CPE-mediated cytolysis was tested along with an appropriate claudin-3- and claudin-4-expressing positive control (i.e., Vero cells, obtained from the American Type Culture Collection), and negative controls which do not express detectable levels of either claudin-3 or claudin-4. As shown in Figure 4, all primary USPC tested were found highly sensitive to CPE-mediated cytolysis. The cytotoxic effect was dose dependent and was positively correlated to the levels of either claudin-3 or claudin-4 expression as tested by RT–PCR in tumor samples. Importantly, although uterine serous tumours demonstrated different sensitivities to CPE exposure, no USPC was found viable after 24 h exposure to CPE at the concentration of 3.3 *μ*g ml^−1^. In contrast, all normal controls tested, including endometrial epithelium, fibroblasts and mononuclear cells lacking claudin-3 or claudin-4, were not affected by CPE (Figure 4).

## DISCUSSION

This report represents the first communication of an investigation involving the genome-wide examination of differences in gene expression between primary USPC and normal endometrial cells (NEC). In this study, we have used short-term primary USPC and NEC cultures (to minimise the risk of a selection bias inherent in any long-term *in vitro* growth) to study differential gene expression in highly enriched populations of epithelial tumour cells and normal cells. We found that hierarchical clustering of the samples and gene expression levels within the samples led to the unambiguous separation of USPC from NEC. We detected 529 genes differentially expressed between USPC and NEC, whose average change in expression level between the two groups was at least five-fold and which were found significant with both WRS test and SAM analysis. The known function of some of these genes may provide insights into the molecular pathogenesis and the highly aggressive biologic behaviour of uterine serous tumours, while others may prove to be useful diagnostic and therapeutic markers against this disease.

For example, the cyclin-dependent kinase inhibitor 2A (*CDKN2A*) gene was found to be the most highly differentially expressed gene in USPC with over 101-fold upregulation relative to NEC. Importantly, the *CDKN2A* gene is a putative oncosuppressor gene encoding two unrelated proteins, both cellular growth inhibitors, in different reading frames (Quelle *et al*, 1995). One is p16, which regulates retinoblastoma protein (pRb)-dependent G1 arrest, and the second is p14ARF, which blocks MDM2-induced p53 degradation, resulting in an increase in p53 levels and consequent cell cycle arrest (Quelle *et al*, 1995). Although loss of p53 function is considered critical for the molecular pathogenesis of USPC (Moll *et al*, 1996), it is only recently that abnormality of the Rb pathway has been suggested to define a subgroup of aggressive endometrial carcinomas with poor prognosis (Salvesen *et al*, 2000). Quantitative RT-PCR analysis of expression of both p16 and p14ARF in our USPC series found extremely high levels of both transcripts, suggesting that the marked overexpression of the *CDKN2A* gene may be attributable to a negative feedback loop due to the loss of function of both pRb and p53 proteins (Moll *et al*, 1996; Salvesen *et al*, 2000). Consistent with this view, an inverse relationship between the expression of p16 and p14ARF proteins and the presence of normal or functional Rb and p53 in human cancer cells has been previously demonstrated (Khleif *et al*, 1996). Thus, our data suggest for the first time that *CDKN2A* gene overexpression may represent a consistent genetic anomaly of USPC secondary to an autoregulatory feedback loop due to disruption of both the p16-CDK4/cyclin D1-pRb pathway and the p14ARF-MDM2-p53 pathway.

Among the several potential therapeutic target gene products identified, genes encoding tight junction (TJ) proteins claudin-3 and claudin-4 were consistently found as two of the most highly upregulated genes in USPC, with over eight- and 12-fold upregulation, respectively, relative to NEC. To the best of our knowledge, claudin-3 and claudin-4 overexpression has not been previously linked to USPC. Although the exact function of claudin-3 and claudin-4 in USPC is still unclear, these proteins have recently been shown to represent the epithelial receptors for CPE (Katahira *et al*, 1997), and to be the only family members of the transmembrane tissue-specific claudin proteins capable of mediating CPE binding and cytolysis. As CPE may trigger a multistep mechanism leading to efficient lysis of mammalian target cells overexpressing claudin-3 and claudin-4 by an increase in membrane permeability resulting in loss of osmotic equilibrium (McClane 1996), CPE-mediated therapy might be a novel, potentially highly effective, strategy for the treatment of USPC refractory to chemotherapy as well as other human tumours overexpressing claudin-3 and/or claudin-4 (Long *et al*, 2001; Michl *et al*, 2001). Mammalian cells that do not express either claudin-3 and/or claudin-4 fail to bind CPE and are not susceptible to CPE cytotoxicity (McClane 1996). Consistent with this view, all primary USPC evaluated, including those found to be resistant to chemotherapy *in vivo* (i.e., USPC-1 and USPC-2, data not shown), were found highly sensitive to CPE-mediated killing *in vitro*. This was in strong contrast with the lack of sensitivity of normal control cells to CPE-mediated cytolysis. Although USPC tumours demonstrated different sensitivity to CPE exposure, no cancer was found viable after 24 h exposure to CPE at a concentration of 3.3 *μ*g ml^−1^, a dose well tolerated by *in vivo* administration of CPE in murine animal models (Wallace *et al*, 1999). More extensive studies will be necessary to evaluate the potential and feasibility of CPE therapy *in vivo* in human patients. Nevertheless, our results provide strong evidence to suggest that CPE-based therapy may have great potential in the treatment of USPC patients refractory to standard treatment modalities. Protein expression data obtained by immunohistochemistry with anti-claudin-4 antibody on an independent set of USPC blocks further support the proposal that claudins may represent therapeutic targets.

The organization of kallikreins, a gene family now consisting of 15 genes that encode for trypsin-like or chymotrypsin-like serine proteases, has been recently elucidated (Diamandis and Yousef, 2002). Serine proteases have well-characterized roles in diverse cellular activities, including blood coagulation, wound healing, digestion, and immune responses, as well as tumour invasion and metastasis (reviewed in Diamandis and Yousef, 2002). Secreted serine proteases such as prostate-specific antigen (PSA) and kallikrein 2 have already found important clinical application as prostate cancer biomarkers (Diamandis and Yousef, 2002). Of interest, kallikrein 6 (also known as zyme/protease M/neurosin) and kallikrein-10 (NES1), two serine proteases recently shown to be present at high levels in the circulation of a subset of ovarian cancer patients (Diamandis *et al*, 2003; Luo *et al*, 2003), were both highly differentially expressed genes in USPC when compared to NEC. Kallikrein 6 and kallikrein 10 overexpression has been shown to correlate with intrinsic resistance to adjuvant chemotherapy and with a poor prognosis in ovarian cancer patients (Diamandis *et al*, 2003; Luo *et al*, 2003). These data are thus consistent with our results showing high expression of kallikrein 6 and kallikrein 10 in USPC, a variant of endometrial carcinoma characterised by an aggressive biologic behaviour and an inborn resistance to chemotherapy (Nicklin and Copeland, 1996). In addition, these results further emphasise the view that kallikrein 6 and kallikrein 10 have the potential to become novel cancer markers for early diagnosis and/or monitoring of USPC, as well as possible immunotherapeutic targets of vaccination strategies against recurrent/refractory serous papillary gynecologic tumours (Cannon *et al*, 2002).

*c-erbB2* gene was found to be one of the most highly differentially expressed gene in USPC with over 14-fold upregulation compared with NEC. Furthermore, the growth factor receptor-bound protein 7 (*GRB7*), a gene tightly linked to c-erbB2 and previously reported coamplified and coexpressed with this gene in several cancer types (Janes *et al*, 1997), was also highly differentially expressed in USPC compared to NEC. These data confirm our recent discovery of a striking overexpression of the *c-erbB2* gene product HER2/neu on 80% of pure USPC (Santin *et al*, 2002). Thus, HER2/neu overexpression may represent a distinctive molecular marker that, in addition to having the potential to facilitate differentiation of USPC from the histologically indistinguishable high-grade serious ovarian tumours (Santin *et al*, 2004), may also provide insights into the disproportionately poor prognosis of USPC patients (Lukes *et al*, 1994; Santin *et al*, 2002, 2004). Previous studies have reported that HER2/neu overexpression in USPC patients may be associated with resistance to chemotherapeutic drugs and shorter survival (Lukes *et al*, 1994). However, high overexpression of the *c-erbB2* gene on USPC provides support for the notion that trastuzumab (Herceptin, Genentech, San Francisco, CA, USA), a humanised anti-HER-2/Neu antibody that is showing great promise for treatment of metastatic breast cancer patients overexpressing HER-2/Neu protein (Slamon *et al*, 2001), may be a novel, potentially highly effective therapy against USPC. Consistent with this view, high sensitivity of USPC to natural killer (NK) cell-mediated antibody-dependent cytotoxicity triggered by anti-HER-2/Neu-specific antibody *in vitro* (Santin *et al*, 2002), as well as clinical responses *in vivo* (Villella *et al*, 2003), have recently been reported with the use of Herceptin in USPC patients.

Several other highly ranked genes have been identified in our USPC gene expression profiling analysis, including membrane-associated protein 17 (MAP17), galanin, urokinase plasminogen activator receptor (UPAR), interleukin-6, forkhead box M1, interleukin-18, dickkopf homolog 1 (DKK1), coagulation factor II (thrombin) receptor-like 1, transforming growth factor, alpha, interleukin 8, topoisomerase (DNA) II alpha, hyaluronan-mediated motility receptor (RHAMM), and secretory leukocyte protease inhibitor (antileukoproteinase). For most of the genes found differentially expressed in our experiments, a correlation with USPC cancer development and progression has not been recognised before. DKK1, for example, has been recently reported by our group to play an important role in the development of osteolytic lesions in multiple myeloma (Tian *et al*, 2003), but its possible role in USPC pathogenesis and/or progression has not been elucidated. For other genes such as UPAR, a glycosyl-phosphatidylinositol-anchored glycoprotein whose role in promoting tumour cell invasion and metastases has been well established in a number of experimental studies, a correlation with high expression in the USPC phenotype has been recently reported (Memarzadeh *et al*, 2002).

A large number of downregulated (at least five-fold) genes in USPC *vs* NEC, such as *transforming growth factor beta receptor III*, *platelet-derived growth factor receptor alpha*, *SEMACAP3*, *ras homolog gene family member I* (*ARHI*), and *DOC1* (Table 3), have been identified in our analysis. Some of these genes encode for widely held tumor suppressor genes such as *SEMACAP3*, *ARHI*, and *DOC1* (Liu and Ganesan, 2002), others for proteins important for tissue homeostasis or that have been previously implicated in apoptosis, proliferation, adhesion, or tissue maintenance. Owing to space limitations, we will not comment further upon the cluster of genes that showed downregulation of the transcripts in invasive tumours.

In conclusion, multiple USPC restricted markers have been identified through our analysis. Most of these genes have not been previously linked with this disease and thus represent novel findings. The identification of HER2/neu and CPE epithelial receptors among others as some of the most highly differentially expressed genes in USPC when compared to NEC suggest that therapeutic strategies targeting HER2/neu by monoclonal antibodies (Villella *et al*, 2003) and claudin-3 and claudin-4 by local and/or systemic administration of CPE (Long *et al*, 2001; Michl *et al*, 2001) may represent novel potentially effective modalities for the treatment of patients harbouring this highly aggressive and chemotherapy-resistant variant of endometrial cancer. The future design and implementation of clinical trials at this regard will ultimately determine the validity of these approaches.

## Figures and Tables

**Table 1:** Characteristics of the patients

**Patient**	**Age**	**Race**	**Stage**
USPC 1	65	White	IV B
USPC 2	75	Afro-American	III C
USPC 3	75	Afro-American	IV A
USPC 4	59	White	IV A
USPC 5	59	White	III C
USPC 6	62	Afro-American	IV B
USPC 7	63	Afro-American	III C
USPC 8	61	Afro-American	III C
USPC 9	78	White	III C
USPC 10	64	Afro-American	IV A

**Table 2:** Upregulated genes expressed at least five-fold higher in USPC compared with NEC

**Probe set**	**Gene symbol**	**SAM**	***P* of WRS**	**Ratio USPC/NEC**	**Title**
1713_s_at	**CDKN2A** a	10.592	0.0027	101.9077377	‘Cyclin-dependent kinase inhibitor 2A (melanoma, p16)’
36288_at	KRTHB1	4.573	0.0027	77.3983986	‘Keratin, hair, basic, 1’
33272_at	SAA1	3.778	0.0063	45.74937337	Serum amyloid A1
41294_at	KRT7	7.173	0.0027	41.46788873	Keratin 7
32154_at	TFAP2A	7.637	0.0027	32.47929396	Transcription factor AP-2 alpha
31610_at	MAP17	3.621	0.0093	30.28302802	Membrane-associated protein 17
408_at	—	4.070	0.0063	30.14111158	—
32821_at	LCN2	5.126	0.0027	27.69975608	Lipocalin 2 (oncogene 24p3)
35174_i_at	EEF1A2	2.840	0.0278	26.80482891	Elongation factor 1 alpha 2
38551_at	**L1CAM**	3.115	0.0196	25.60938089	‘L1 cell adhesion molecule
38249_at	VGLL1	5.274	0.0027	24.69495091	Vestigial like 1 (Drosophila)
35879_at	GAL	5.594	0.0027	23.48953559	Galanin
36838_at	KLK10	3.455	0.0136	23.17518549	Kallikrein 10
38299_at	IL6	3.630	0.0041	19.05873079	‘Interleukin 6 (interferon, beta 2)’
38051_at	MAL	4.878	0.0041	17.51555106	‘mal, T-cell differentiation protein’
41469_at	PI3	2.854	0.0063	16.90464558	‘Protease inhibitor 3, skin-derived (SKALP)’
40412_at	PTTG1	5.218	0.0027	16.61222352	Pituitary tumour-transforming 1
1886_at	WNT7A	3.544	0.0196	16.11519778	‘Wingless-type MMTV integration site family, member 7A’
33128_s_at	CST6	4.222	0.0136	15.97856318	Cystatin E/M
38414_at	CDC20	7.317	0.0027	15.64601435	CDC20 cell division cycle 20 homolog (*S. cerevisiae*)
34012_at	KRTHA4	2.411	0.0278	15.37247475	‘Keratin, hair, acidic, 4’
37554_at	KLK6	3.785	0.0093	15.23781352	‘Kallikrein 6 (neurosin, zyme)’
1802_s_at	**ERBB2**	2.566	0.0136	14.52012028	‘v-erb-b2’
41060_at	CCNE1	6.092	0.0027	14.16647068	Cyclin E1
36837_at	KIF2C	6.130	0.0027	14.1328483	Kinesin family member 2C
34213_at	KIBRA	5.301	0.0027	13.27228177	KIBRA protein
1651_at	UBE2C	5.554	0.0027	12.87617243	Ubiquitin-conjugating enzyme E2C
35276_at	**CLDN4**	6.381	0.0027	12.74825421	Claudin 4
36990_at	UCHL1	4.623	0.0027	12.30505908	Ubiquitin carboxyl-terminal esterase L1
35977_at	DKK1	4.495	0.0041	12.25382636	Dickkopf homolog 1 (*Xenopus laevis*)
36113_s_at	TNNT1	4.072	0.0027	11.93824813	‘Troponin T1, skeletal, slow’
2011_s_at	BIK	3.451	0.0063	11.66959681	BCL2-interacting killer (apoptosis-inducing)
543_g_at	CRABP1	3.193	0.0093	11.55494382	Cellular retinoic acid binding protein 1
34852_g_at	STK6	6.225	0.0027	11.51812047	Serine/threonine kinase 6
33483_at	NMU	4.094	0.0027	11.42057993	Neuromedin U
39109_at	TPX2	6.162	0.0027	11.29208457	‘TPX2, microtubule-associated protein homologue’
37018_at	HIST1H1C	2.270	0.0278	10.74270622	‘Histone 1, H1c’
1165_at	IL18	3.221	0.0041	10.65596528	Interleukin 18 (interferon-gamma-inducing factor)
36477_at	TNNI3	2.867	0.0136	10.61101382	‘Troponin I, cardiac’
572_at	TTK	3.720	0.0093	9.723902052	TTK protein kinase
31542_at	FLG	2.622	0.0196	9.600831601	Filaggrin
35937_at	MICB	4.238	0.0093	9.460109582	MHC class I polypeptide-related sequence B
36155_at	SPOCK2	2.278	0.0278	9.216570003	‘Sparc/osteonectin, cwcv and kazal-like domains proteoglycan (testican) 2’
32186_at	SLC7A5	4.149	0.0063	9.121679665	‘Solute carrier family 7 (cationic amino-acid transporter, *y*+ system), member 5’
35766_at	KRT18	5.933	0.0027	9.01220054	Keratin 18
35822_at	BF	3.267	0.0063	8.952514469	‘B-factor, properdin’
35714_at	PDXK	6.550	0.0027	8.898191704	‘Pyridoxal (pyridoxine, vitamin B6) kinase’
1369_s_at	—	2.679	0.0196	8.773380878	—
40079_at	RAI3	4.516	0.0063	8.626843209	Retinoic acid induced 3
37168_at	LAMP3	2.838	0.0136	8.616807346	Lysosomal-associated membrane protein 3
39704_s_at	HMGA1	5.322	0.0027	8.597894471	High-mobility group AT-hook 1
1887_g_at	WNT7A	3.003	0.0196	8.491813649	‘Wingless-type MMTV integration site family, member 7A’
36929_at	LAMB3	5.770	0.0027	8.354149098	‘Laminin, beta 3’
527_at	CENPA	6.126	0.0027	8.32992789	‘Centromere protein A, 17 kDa’
41081_at	BUB1	4.883	0.0027	8.213759056	BUB1 budding uninhibited by benzimidazoles 1 homologue
885_g_at	ITGA3	4.447	0.0027	8.20660555	‘Integrin, alpha 3 (antigen CD49C, alpha 3 subunit of VLA-3 receptor)’
2021_s_at	CCNE1	4.399	0.0041	8.199388463	Cyclin E1
33904_at	**CLDN3**	3.296	0.0136	8.020010794	Claudin 3
33730_at	RAI3	4.648	0.0041	7.923899439	Retinoic acid induced 3
34736_at	CCNB1	5.078	0.0063	7.896644626	Cyclin B1
757_at	ANXA2	3.514	0.0063	7.870466864	Annexin A2
910_at	TK1	3.934	0.0093	7.869091533	‘Thymidine kinase 1, soluble’
34851_at	STK6	4.491	0.0041	7.764803777	Serine/threonine kinase 6
34703_f_at	—	2.275	0.0278	7.710260816	—
34715_at	FOXM1	5.318	0.0027	7.659023602	Forkhead box M1
38971_r_at	TNIP1	6.800	0.0027	7.595036872	TNFAIP3 interacting protein 1
32263_at	CCNB2	4.246	0.0063	7.578513543	Cyclin B2
1680_at	**GRB7**	4.013	0.0027	7.471384928	Growth factor receptor-bound protein 7
38247_at	F2RL1	3.185	0.0093	7.432476326	Coagulation factor II (thrombin) receptor-like 1
160025_at	TGFA	6.088	0.0027	7.355344272	‘Transforming growth factor, alpha’
1945_at	CCNB1	5.298	0.0041	7.291039832	Cyclin B1
31792_at	ANXA3	4.873	0.0041	7.266892828	Annexin A3
182_at	ITPR3	5.432	0.0027	7.172450367	‘Inositol 1,4,5-triphosphate receptor, type 3’
1117_at	CDA	2.937	0.0093	7.114518646	Cytidine deaminase
902_at	EPHB2	5.186	0.0027	7.065363569	EphB2
634_at	PRSS8	5.216	0.0041	7.001894703	‘Protease, serine, 8 (prostasin)’
41169_at	PLAUR	3.982	0.0063	7.00139089	‘Plasminogen activator, urokinase receptor’
33203_s_at	FOXD1	3.464	0.0093	6.989749222	Forkhead box D1
40095_at	CA2	4.285	0.0027	6.946396937	Carbonic anhydrase II
38940_at	AD024	5.065	0.0041	6.928406028	AD024 protein
34348_at	SPINT2	6.263	0.0027	6.877224695	‘Serine protease inhibitor, Kunitz type, 2’
33933_at	WFDC2	3.344	0.0136	6.820073691	WAP four-disulphide core domain 2
35281_at	LAMC2	3.347	0.0093	6.7580474	‘Laminin, gamma 2’
349_g_at	KIFC1	5.275	0.0041	6.700913018	Kinesin family member C1
33218_at	ERBB2	2.710	0.0027	6.615105998	‘v-erb-b2 erythroblastic leukemia viral oncogene homolog 2, neuro/glioblastoma derived oncogene homolog (avian)’
38881_i_at	TRIM16	3.001	0.0196	6.506893575	Tripartite motif-containing 16
1536_at	CDC6	4.666	0.0041	6.463305623	CDC6 cell division cycle 6 homolog (*S. cerevisiae*)
38482_at	CLDN7	4.931	0.0041	6.409117877	Claudin 7
40697_at	CCNA2	3.396	0.0093	6.40768505	Cyclin A2
41688_at	TM4SF11	4.390	0.0027	6.366861533	Transmembrane 4 superfamily member 11 (plasmolipin)
38158_at	ESPL1	6.007	0.0027	6.225688779	Extra spindle poles like 1 (*S. cerevisiae*)
38474_at	CBS	3.380	0.0093	6.212078913	Cystathionine-beta-synthase
36483_at	GALNT3	3.890	0.0041	6.181109111	UDP-*N*-acetyl-alpha-D-galactosamine:polypeptide *N*-acetylgalactosaminyltransferase 3 (GalNAc-T3)
35372_r_at	IL8	2.360	0.0278	6.133149591	Interleukin 8
41585_at	KIAA0746	4.436	0.0027	6.092207586	KIAA0746 protein
36832_at	B3GNT3	5.457	0.0027	5.941291793	‘UDP-GlcNAc:betaGal beta-1,3-*N*-acetylglucosaminyltransferase 3’
1107_s_at	G1P2	3.938	0.0063	5.923287019	‘Interferon, alpha-inducible protein (clone IFI-15K)’
35207_at	SCNN1A	3.076	0.0136	5.920739634	‘Sodium channel, nonvoltage-gated 1 alpha’
36863_at	HMMR	2.830	0.0196	5.905038013	Hyaluronan-mediated motility receptor (RHAMM)
38631_at	TNFAIP2	4.924	0.0027	5.897745642	‘Tumour necrosis factor, alpha-induced protein 2’
36813_at	TRIP13	5.666	0.0027	5.870351247	Thyroid hormone receptor interactor 13
41048_at	PMAIP1	3.490	0.0062	5.853172336	Phorbol-12-myristate-13-acetate-induced protein 1
2084_s_at	ETV4	3.743	0.0093	5.798002338	‘ets variant gene 4 (E1A enhancer binding protein, E1AF)’
33245_at	MAPK13	3.775	0.0136	5.766618762	Mitogen-activated protein kinase 13
37347_at	CKS1B	5.543	0.0027	5.762817533	CDC28 protein kinase regulatory subunit 1B
34282_at	NFE2L3	2.668	0.0136	5.734907375	Nuclear factor (erythroid-derived 2)-like 3
330_s_at	—	4.026	0.0041	5.726752495	—
41732_at	na	6.920	0.0027	5.706487141	Similar to My016 protein
1516_g_at	—	6.726	0.0027	5.63870137	—
904_s_at	TOP2A	3.419	0.0063	5.634251452	Topoisomerase (DNA) II alpha 170 kDa
36041_at	EXO1	4.971	0.0027	5.59235892	Exonuclease 1
33143_s_at	SLC16A3	4.007	0.0063	5.56591457	‘Solute carrier family 16 (monocarboxylic acid transporters), member 3’
37228_at	PLK	4.501	0.0041	5.564532365	Polo-like kinase (Drosophila)
1854_at	MYBL2	4.117	0.0063	5.54317592	v-myb myeloblastosis viral oncogene homolog (avian)-like 2
40407_at	KPNA2	4.189	0.0041	5.51635645	‘Karyopherin alpha 2 (RAG cohort 1, importin alpha 1)’
33282_at	LAD1	3.904	0.0063	5.509367036	Ladinin 1
40145_at	TOP2A	3.307	0.0093	5.48127065	Topoisomerase (DNA) II alpha 170 kDa
1100_at	IRAK1	5.530	0.0027	5.470162749	Interleukin-1 receptor-associated kinase 1
37883_i_at	AF038169	3.160	0.0027	5.460495655	Hypothetical protein AF038169
37343_at	ITPR3	5.258	0.0027	5.449013729	‘Inositol 1,4,5-triphosphate receptor, type 3’
31598_s_at	GALE	4.647	0.0027	5.442955253	‘Galactose-4-epimerase, UDP-’
889_at	ITGB8	2.744	0.0093	5.370592815	‘Integrin, beta 8’
37558_at	IMP-3	3.123	0.0093	5.364127468	IGF-II mRNA-binding protein 3
32715_at	VAMP8	5.686	0.0027	5.352873419	Vesicle-associated membrane protein 8 (endobrevin)
36312_at	SERPINB8	3.611	0.0027	5.327343554	‘Serine (or cysteine) proteinase inhibitor, clade B (ovalbumin), member 8’
37210_at	INA	3.551	0.0063	5.307526088	‘Internexin neuronal intermediate filament protein, alpha’
35699_at	BUB1B	3.665	0.0196	5.279075308	BUB1 budding uninhibited by benzimidazoles 1 homolog beta (yeast)
32787_at	ERBB3	2.658	0.0041	5.247404657	v-erb-b2 erythroblastic leukemia viral oncogene homolog 3 (avian)
32275_at	SLPI	3.726	0.0041	5.221163981	Secretory leukocyte protease inhibitor (antileukoproteinase)
893_at	E2-EPF	3.775	0.0063	5.196412396	Ubiquitin carrier protein
41583_at	FEN1	5.481	0.0027	5.196005796	Flap structure-specific endonuclease 1
41781_at	PPFIA1	4.113	0.0027	5.194931774	‘Protein tyrosine phosphatase, receptor type, f polypeptide (PTPRF), interacting protein (liprin), alpha 1’
40726_at	KIF11	2.941	0.0093	5.1806793	Kinesin family member 11
41400_at	TK1	4.246	0.0093	5.167172588	‘Thymidine kinase 1, soluble’
41409_at	C1orf38	3.109	0.0063	5.100239097	Chromosome 1 open reading frame 38
40425_at	EFNA1	2.738	0.0196	5.067718102	Ephrin-A1
32081_at	CIT	6.162	0.0027	5.043567722	‘Citron (rho-interacting, serine/threonine kinase 21)’
1108_s_at	EPHA1	4.864	0.0027	5.040980858	EphA1
33338_at	STAT1	3.275	0.0063	5.029498048	‘Signal transducer and activator of transcription 1, 91 kDa’

a Genes selected for further analysis are identified with bold letters.

**Table 3:** Upregulated genes expressed at least 10-fold higher in NEC compared with USPC

**Probe set**	**Gene symbol**	**SAM**	***P* of WRS**	**Ratio NEC/USPC**	**Title**
774_g_at	MYH11	17.611	0.0027	1014.968759	‘Myosin, heavy polypeptide 11, smooth muscle’
773_at	MYH11	14.265	0.0027	968.0223497	‘Myosin, heavy polypeptide 11, smooth muscle’
32582_at	MYH11	11.277	0.0027	212.2458648	‘Myosin, heavy polypeptide 11, smooth muscle’
36681_at	APOD	11.714	0.0027	137.3140116	Apolipoprotein D
1501_at	IGF1	8.321	0.0027	128.0651104	Insulin-like growth factor 1 (somatomedin C)
39325_at	EBAF	8.177	0.0027	123.064609	‘Endometrial bleeding associated factor)’
767_at	—	12.707	0.0027	119.5409103	—
40398_s_at	MEOX2	10.182	0.0027	114.9489897	Mesenchyme homeo box 2
40776_at	DES	9.738	0.0027	109.416397	Desmin
1197_at	ACTG2	5.861	0.0027	96.65715109	‘Actin, gamma 2, smooth muscle, enteric’
36627_at	SPARCL1	7.724	0.0027	93.89302842	‘SPARC-like 1 (mast9, hevin)’
37407_s_at	MYH11	11.893	0.0027	88.70362054	‘Myosin, heavy polypeptide 11, smooth muscle’
39673_i_at	ECM2	8.459	0.0027	82.52353674	‘Extracellular matrix protein 2, female organ and adipocyte specific’
39701_at	PEG3	7.645	0.0027	73.87997223	Paternally expressed gene 3
39066_at	MFAP4	9.383	0.0027	68.84723295	Microfibrillar-associated protein 4
38737_at	IGF1	6.341	0.0027	65.13666715	Insulin-like growth factor 1 (somatomedin C)
35730_at	ADH1B	9.759	0.0027	64.94146631	‘Alcohol dehydrogenase IB (class I), beta polypeptide’
41124_r_at	ENPP2	7.007	0.0027	53.43118221	Ectonucleotide pyrophosphatase/phosphodiesterase 2 (autotaxin)
36749_at	CPA3	9.096	0.0027	53.07347991	Carboxypeptidase A3 (mast cell)
39616_at	PTGER3	10.188	0.0027	52.65326101	Prostaglandin E receptor 3 (subtype EP3)
33440_at	TCF8	7.220	0.0027	49.54168001	Transcription factor 8
32666_at	CXCL12	7.168	0.0027	49.2359637	Chemokine (C–X–C motif) ligand 12
38734_at	PLN	9.182	0.0027	47.86730799	Phospholamban
34203_at	CNN1	5.051	0.0027	47.0209722	‘Calponin 1, basic, smooth muscle’
37576_at	PCP4	12.468	0.0027	45.45542357	Purkinje cell protein 4
36834_at	MOXD1	9.736	0.0027	44.20917891	‘Monooxygenase, DBH-like 1’
37247_at	TCF21	9.810	0.0027	42.40051895	Transcription factor 21
37701_at	RGS2	7.408	0.0027	42.29038464	‘Regulator of G-protein signalling 2, 24 kDa’
779_at	COL4A6	5.840	0.0027	41.96325597	‘Collagen, type IV, alpha 6’
37394_at	C7	9.835	0.0027	40.72326307	Complement component 7
36533_at	PTGIS	6.097	0.0027	39.10052985	Prostaglandin I2 (prostacyclin) synthase
32689_s_at	—	6.178	0.0027	36.96897988	—
32905_s_at	TPSB2	6.873	0.0027	36.85974711	Tryptase beta 2
33240_at	SEMACAP3	4.814	0.0027	36.54583796	Likely ortholog of mouse semaF cytoplasmic domain associated protein 3
1466_s_at	FGF7	8.628	0.0027	35.60413415	Fibroblast growth factor 7
32686_at	PTGER3	7.397	0.0027	35.31357002	Prostaglandin E receptor 3 (subtype EP3)
39690_at	ALP	6.951	0.0027	33.76045029	Alpha-actinin-2-associated LIM protein
38427_at	COL15A1	7.196	0.0027	32.85391153	‘Collagen, type XV, alpha 1’
39939_at	COL4A6	6.846	0.0027	32.53815505	‘Collagen, type IV, alpha 6’
37630_at	NRLN1	5.896	0.0027	31.03030255	Neuralin 1
35717_at	ABCA8	7.587	0.0027	30.63819827	‘ATP-binding cassette, subfamily A, member 8’
1709_g_at	MAPK10	6.522	0.0027	30.54331363	Mitogen-activated protein kinase 10
35679_s_at	DPP6	5.899	0.0027	30.33959319	Dipeptidylpeptidase 6
35740_at	EMILIN1	7.445	0.0027	29.45261895	Elastin microfibril interfacer 1
41123_s_at	ENPP2	6.597	0.0027	29.29614059	Ectonucleotide pyrophosphatase/phosphodiesterase 2 (autotaxin)
755_at	ITPR1	8.211	0.0027	29.02124207	‘Inositol 1,4,5-triphosphate receptor, type 1’
32847_at	MYLK	5.388	0.0027	28.22518538	‘Myosin, light polypeptide kinase’
38001_at	CACNA1C	5.740	0.0027	27.31347302	‘Calcium channel, alpha 1C subunit’
37279_at	GEM	9.925	0.0027	26.56613192	GTP-binding protein overexpressed in skeletal muscle
36396_at	—	5.901	0.0027	26.32457357	*Homo sapiens* mRNA; cDNA DKFZp586N2020 (from clone DKFZp586N2020)
41137_at	PPP1R12B	12.043	0.0027	25.97089935	‘Protein phosphatase 1, regulatory subunit 12B’
41405_at	SFRP4	10.077	0.0027	23.90433923	Secreted frizzled-related protein 4
40775_at	ITM2A	6.152	0.0027	23.83083993	Integral membrane protein 2A
38059_g_at	DPT	6.508	0.0027	23.76376781	Dermatopontin
41504_s_at	MAF	5.331	0.0027	23.65323618	v-maf
1596_g_at	TEK	4.542	0.0027	23.37422615	‘TEK tyrosine kinase, endothelial (venous malformations, multiple cutaneous and mucosal)’
914_g_at	ERG	6.105	0.0027	22.63829292	v-ets erythroblastosis virus E26 oncogene like
34283_at	—	5.390	0.0041	22.19917455	‘*Homo sapiens*, clone IMAGE:4791553, mRNA’
34388_at	COL14A1	9.452	0.0027	21.48165459	‘Collagen, type XIV, alpha 1 (undulin)’
38994_at	SOCS2	6.600	0.0027	21.47663968	Suppressor of cytokine signaling 2
36065_at	LDB2	5.790	0.0027	21.19321308	LIM domain binding 2
40230_at	FRZB	5.962	0.0027	21.00467856	Frizzled-related protein
33790_at	CCL15	9.017	0.0027	20.5510028	Chemokine (C–C motif) ligand 15
33890_at	RGS5	7.347	0.0027	20.2203337	Regulator of G-protein signalling 5
36513_at	MAGP2	4.385	0.0041	20.10734871	Microfibril-associated glycoprotein-2
32526_at	JAM3	6.129	0.0027	19.89641568	Junctional adhesion molecule 3
32687_s_at	PTGER3	5.902	0.0027	19.7194642	Prostaglandin E receptor 3 (subtype EP3)
35638_at	CBFA2T1	10.470	0.0027	19.57233853	‘Core-binding factor, runt domain, alpha subunit 2; translocated to, 1; cyclin D-related’
34637_f_at	ADH1A	6.291	0.0027	19.20847669	‘Alcohol dehydrogenase 1A (class I)’
34675_at	SBLF	5.400	0.0027	19.04463285	Stoned B-like factor
38351_at	—	5.899	0.0026	18.69131208	*Homo sapiens* mRNA; cDNA DKFZp586L0120 (from clone DKFZp586L0120)
1182_at	PLCL1	4.047	0.0027	18.50958764	Phospholipase C-like 1
39681_at	ZNF145	6.266	0.0027	18.41823634	‘Zinc-finger protein 145 (Kruppel-like, expressed in promyelocytic leukemia)’
1708_at	MAPK10	6.336	0.0027	18.31250756	Mitogen-activated protein kinase 10
37765_at	LMOD1	7.586	0.0027	18.11624265	Leiomodin 1 (smooth muscle)
1678_g_at	IGFBP5	4.237	0.0041	17.88349387	Insulin-like growth factor binding protein 5
35358_at	TENC1	9.243	0.0027	17.68277053	Tensin-like C1 domain-containing phosphatase
33442_at	KIAA0367	6.830	0.0027	17.58195512	KIAA0367 protein
37249_at	PDE8B	5.035	0.0027	17.25176904	Phosphodiesterase 8B
33834_at	CXCL12	7.736	0.0027	17.10893345	Chemokine (C–X–C motif) ligand 12 (stromal cell-derived factor 1)
35324_at	SLIT3	5.157	0.0027	17.08441711	Slit homolog 3 (*Drosophila*)
37015_at	ALDH1A1	4.941	0.0027	16.83181709	‘Aldehyde dehydrogenase 1 family, member A1’
39266_at	—	6.237	0.0027	16.80733058	*Homo sapiens* clone 24405 mRNA sequence
33462_at	GPR105	4.907	0.0027	16.49634139	G protein-coupled receptor 105
32488_at	COL3A1	3.928	0.0027	16.44993439	‘Collagen, type III, alpha 1 (Ehlers-Danlos syndrome type IV, autosomal dominant)’
483_g_at	CDH13	3.755	0.0027	16.43857351	‘Cadherin 13, H-cadherin (heart)’
38026_at	FBLN1	5.350	0.0027	16.06497318	Fibulin 1
1909_at	BCL2	7.356	0.0027	16.01160367	B-cell CLL/lymphoma 2
36245_at	HTR2B	4.698	0.0027	15.77119379	5-Hydroxytryptamine (serotonin) receptor 2B
32057_at	LRRC17	5.470	0.0027	15.6891271	Leucine-rich repeat containing 17
39544_at	DMN	5.361	0.0027	15.66465434	Desmuslin
37112_at	C6orf32	4.720	0.0027	15.65975413	Chromosome 6 open reading frame 32
36733_at	FLJ32389	4.442	0.0027	15.64273348	Hypothetical protein FLJ32389
1319_at	DDR2	5.400	0.0027	15.5817123	‘Discoidin domain receptor family, member 2’
38057_at	DPT	8.479	0.0027	15.51358362	Dermatopontin
40358_at	GLI3	6.650	0.0027	15.11249492	GLI-Kruppel family member GLI3 (Greig cephalopolysyndactyly syndrome)
38627_at	HLF	4.947	0.0027	14.89249894	Hepatic leukemia factor
1731_at	PDGFRA	7.050	0.0027	14.84422948	‘Platelet-derived growth factor receptor, alpha polypeptide’
31897_at	DOC1	4.664	0.0027	14.80084961	Downregulated in ovarian cancer 1
2073_s_at	CDH13	5.076	0.0027	14.79118507	‘Cadherin 13, H-cadherin (heart)’
38577_at	—	7.283	0.0027	14.78670234	‘Clone DT1P1B6 mRNA, CAG repeat region’
38004_at	CSPG4	6.072	0.0027	14.40637488	Chondroitin sulphate proteoglycan 4
32109_at	FXYD1	7.333	0.0027	14.33463902	Phospholemman
34853_at	FLRT2	9.834	0.0027	14.33080432	Fibronectin leucine-rich transmembrane protein 2
38298_at	KCNMB1	7.046	0.0027	14.25816751	‘Potassium large-conductance calcium-activated channel, subfamily M, beta member 1’
41245_at	GDF10	6.643	0.0027	14.21467179	Growth differentiation factor 10
38322_at	GAGEC1	6.215	0.0027	14.10508119	‘G antigen, family C, 1’
1198_at	EDNRB	4.899	0.0027	14.08196951	Endothelin receptor type B
41505_r_at	MAF	4.528	0.0027	14.0059518	v-maf
36976_at	CDH11	3.692	0.0027	14.00329993	‘Cadherin 11, type 2, OB-cadherin (osteoblast)’
32688_at	PTGER3	4.998	0.0027	13.68791213	Prostaglandin E receptor 3 (subtype EP3)
1575_at	ABCB1	6.210	0.0027	13.64196645	‘ATP-binding cassette, subfamily B’
32778_at	ITPR1	4.914	0.0027	13.60359986	‘Inositol 1,4,5-triphosphate receptor, type 1’
40737_at	KCNMA1	5.642	0.0027	13.60011778	‘potassium large-conductance calcium-activated channel, subfamily M, alpha member 1’
36569_at	TNA	11.512	0.0027	13.14186685	Tetranectin (plasminogen-binding protein)
1897_at	TGFBR3	4.922	0.0041	12.99722509	‘Transforming growth factor, beta receptor III (betaglycan, 300 kDa)’
2087_s_at	CDH11	4.345	0.0027	12.96131894	‘Cadherin 11, type 2, OB-cadherin (osteoblast)’
34820_at	PTN	4.413	0.0041	12.94626537	‘Pleiotrophin (heparin binding growth factor 8, neurite growth-promoting factor 1)’
743_at	NAP1L3	7.859	0.0027	12.91061134	Nucleosome assembly protein 1-like 3
1507_s_at	EDNRA	4.319	0.0041	12.88466839	Endothelin receptor type A
38995_at	CLDN5	12.156	0.0027	12.86636376	Claudin 5 (transmembrane protein deleted in velocardiofacial syndrome)
1147_at	—	7.084	0.0027	12.69931721	—
1954_at	KDR	6.077	0.0027	12.6174344	Kinase insert domain receptor (a type III receptor tyrosine kinase)
38786_at	—	5.676	0.0027	12.61170471	*Homo sapiens* mRNA full-length insert cDNA clone EUROIMAGE 248114
40757_at	GZMA	4.575	0.0041	12.56281091	‘Granzyme A (granzyme 1, cytotoxic T-lymphocyte-associated serine esterase 3)’
36695_at	na	5.776	0.0027	12.53905775	Similar to hypothetical protein 6720478C22
32052_at	HBB	5.107	0.0063	12.52262761	‘Haemoglobin, beta’
37446_at	GASP	8.227	0.0027	12.39360585	G protein-coupled receptor-associated protein
38038_at	LUM	4.462	0.0027	12.34757673	Lumican
32889_at	RPIB9	6.337	0.0027	12.34540513	Rap2-binding protein 9
35234_at	RECK	4.476	0.0027	12.33793353	Reversion-inducing-cysteine-rich protein
661_at	GAS1	4.648	0.0027	12.32556384	Growth arrest-specific 1
41195_at	LPP	6.292	0.0027	12.26636801	LIM domain containing preferred translocation partner in lipoma
32664_at	RNASE4	6.395	0.0027	11.89680804	‘Ribonuclease, RNase A family, 4’
39038_at	FBLN5	4.325	0.0027	11.89440528	Fibulin 5
40693_at	KCNB1	4.952	0.0041	11.84773944	‘Potassium voltage-gated channel, Shab-related subfamily, member 1’
38052_at	F13A1	5.346	0.0027	11.69181717	‘Coagulation factor XIII, A1 polypeptide’
35220_at	ENPEP	4.200	0.0027	11.66308093	Glutamyl aminopeptidase (aminopeptidase A)
37512_at	RODH	5.112	0.0027	11.65031202	3-Hydroxysteroid epimerase
103_at	THBS4	5.114	0.0027	11.6063075	Thrombospondin 4
36156_at	AQP1	6.927	0.0027	11.6016663	‘Aquaporin 1’
39593_at	FGL2	5.176	0.0027	11.38223584	Fibrinogen-like 2
33248_at	HOXA11	6.555	0.0027	11.34828098	Homeo box A11
41420_at	IGFBP5	4.166	0.0041	11.32365037	Insulin-like growth factor binding protein 5
35644_at	HEPH	6.666	0.0027	11.26324425	Hephaestin
39646_at	CACNB2	4.719	0.0027	11.25017414	‘Calcium channel, voltage-dependent, beta 2 subunit’
35333_r_at	DVS27	4.403	0.0027	11.23759398	DVS27-related protein
33182_at	—	4.990	0.0027	11.20258783	*Homo sapiens* mRNA full-length insert cDNA clone EUROIMAGE 1630957
34303_at	FLJ90798	4.909	0.0027	11.10458088	Hypothetical protein FLJ90798
33756_at	AOC3	5.186	0.0027	11.09776026	‘Amine oxidase, copper containing 3 (vascular adhesion protein 1)’
37710_at	MEF2C	7.485	0.0027	11.03832245	‘MADS box transcription enhancer factor 2, polypeptide C (myocyte enhancer factor 2C)’
35680_r_at	DPP6	3.783	0.0027	10.88151987	Dipeptidylpeptidase 6
40126_at	—	4.620	0.0027	10.87424764	—
31831_at	SMTN	5.712	0.0027	10.84917408	Smoothelin
234_s_at	PTN	5.660	0.0041	10.803161	‘Pleiotrophin (heparin binding growth factor 8, neurite growth-promoting factor 1)’
36939_at	GPM6A	4.981	0.0027	10.76644676	Glycoprotein M6A
41158_at	PLP1	4.879	0.0027	10.76462278	‘Proteolipid protein 1 (Pelizaeus-Merzbacher disease, spastic paraplegia 2, uncomplicated)’
41839_at	GAS1	4.401	0.0027	10.75375673	Growth arrest-specific 1
1186_at	GDF10	4.165	0.0063	10.74677086	Growth differentiation factor 10
35404_at	TACR2	4.416	0.0063	10.67366905	Tachykinin receptor 2
160023_at	WNT2	5.424	0.0027	10.67077677	Wingless-type MMTV integration site family member 2
34561_at	MS4A2	6.380	0.0027	10.6336763	‘Membrane-spanning 4-domains, subfamily A, member 2 (Fc fragment of IgE, high affinity I, receptor for; beta polypeptide)’
36280_at	GZMK	4.739	0.0027	10.5607572	‘Granzyme K’
35668_at	RAMP1	3.430	0.0027	10.4886967	Receptor (calcitonin) activity modifying protein 1
32521_at	SFRP1	4.488	0.0027	10.43051698	Secreted frizzled-related protein 1
1975_s_at	IGF1	7.381	0.0027	10.34307322	Insulin-like growth factor 1 (somatomedin C)
37671_at	LAMA4	5.094	0.0027	10.34084543	‘Laminin, alpha 4’
40013_at	CLIC2	5.388	0.0027	10.2736905	Chloride intracellular channel 2
32782_r_at	BPAG1	4.597	0.0027	10.2197061	‘Bullous pemphigoid antigen 1, 230/240 kDa’
36918_at	GUCY1A3	4.344	0.0027	10.18606373	‘Guanylate cyclase 1, soluble, alpha 3’
32239_at	MATN2	6.197	0.0027	10.14895891	Matrilin 2
36503_at	CCL21	6.812	0.0027	10.09354903	Chemokine (C–C motif) ligand 21
38508_s_at	TNXB	8.826	0.0027	10.0794719	Tenascin XB
35146_at	TGFB1I1	5.562	0.0027	10.0680837	Transforming growth factor beta 1
38653_at	PMP22	6.085	0.0027	10.04665889	Peripheral myelin protein 22
342_at	ENPP1	5.008	0.0041	10.0434301	Ectonucleotide pyrophosphatase/phosphodiesterase
32826_at	ENTPD1	6.423	0.0027	10.01186286	Ectonucleoside triphosphate diphosphohydrolase 1
40318_at	DNCI1	3.348	0.0063	10.00454416	‘Dynein, cytoplasmic, intermediate polypeptide 1’

**Table 4:** Claudin-4 staining

**Patient**	**Claudin-4 positivity**
NEC 1	1+
NEC 2	1+
NEC 3	1+
NEC 4	1+
NEC 5	1+
USPC 1	3+
USPC 2	3+
USPC 3	3+
USPC 4	2+
USPC 5	3+
USPC 6	2+
USPC 7	3+
USPC 8	1+
USPC 9	3+
USPC 10	3+
USPC 11	3+
USPC 12	3+
USPC 13	1+
USPC 14	2+
USPC 15	3+
USPC 16	2+
USPC 17	3+
USPC 18	2+
